# Evaluation of drug-drug interaction of lusutrombopag, a thrombopoietin receptor agonist, via metabolic enzymes and transporters

**DOI:** 10.1007/s00228-020-02960-7

**Published:** 2020-07-14

**Authors:** Takayuki Katsube, Yuji Inoue, Takahiro Fukuhara, Takeshi Kano, Toshihiro Wajima

**Affiliations:** 1 Clinical Pharmacology & Pharmacokinetics, Shionogi & Co., Ltd., 1-4, Shibata 1-chome, Kita-ku, Osaka, 530-0012 Japan; 2 Drug Metabolism & Pharmacokinetics Department, Shionogi & Co., Ltd., 1-1, Futaba-cho, 3-chome, Toyonaka, 561-0825 Japan; 3 Clinical Research Department, Shionogi & Co., Ltd., 1-4, Shibata 1-chome, Kita-ku, Osaka, 530-0012 Japan

**Keywords:** Lusutrombopag, Drug interaction, Pharmacokinetics, Metabolic enzyme, Transporter

## Abstract

**Purpose:**

Drug-drug interaction (DDI) potentials of lusutrombopag, a thrombopoietin receptor agonist, on the activity of cytochrome P450 (CYP) 3A and of cyclosporine, which inhibits P-glycoprotein and breast cancer resistance protein, on lusutrombopag pharmacokinetics were assessed via clinical studies and physiologically based pharmacokinetic (PBPK) modeling.

**Methods:**

The effect of lusutrombopag on midazolam (a CYP3A probe substrate) pharmacokinetics was assessed in 15 healthy subjects receiving a single midazolam 5-mg dose with or without coadministration of lusutrombopag 0.75 mg for 6 days (first dose: 1.5-mg dose). The effect of cyclosporine on lusutrombopag pharmacokinetics was assessed in 16 healthy subjects receiving a single lusutrombopag 3-mg dose with or without a single cyclosporine 400- to 600-mg dose. PBPK modeling was employed to extrapolate the effect of lusutrombopag at the clinical dose (3 mg once daily) on midazolam pharmacokinetics.

**Results:**

In the clinical study, mean ratios (90% confidence intervals [CIs]) of with/without lusutrombopag for maximum plasma concentration (*C*_max_) and area under the plasma concentration-time curve (AUC) of midazolam were 1.01 (0.908–1.13) and 1.04 (0.967–1.11), respectively, indicating no effect of lusutrombopag on midazolam pharmacokinetics. PBPK modeling suggested no effect of lusutrombopag at the clinical dose on midazolam pharmacokinetics. Mean ratios (90% CIs) of with/without cyclosporine for lusutrombopag *C*_max_ and AUC were 1.18 (1.11–1.24) and 1.19 (1.13–1.25), respectively, indicating a slight increase in lusutrombopag exposure.

**Conclusions:**

In consideration with in vitro data, the in vivo and in silico results suggested no clinically significant DDI potential of lusutrombopag with other medical products via metabolic enzymes and transporters.

**Electronic supplementary material:**

The online version of this article (10.1007/s00228-020-02960-7) contains supplementary material, which is available to authorized users.

## Introduction

Lusutrombopag (S-888711) is a small-molecule thrombopoietin (TPO) receptor agonist developed by Shionogi & Co., Ltd. (Osaka, Japan). Lusutrombopag promotes thrombocytopoiesis in the same fashion as endogenous TPO. Thrombocytopenia commonly develops in patients with chronic liver disease (CLD) [1]. Lusutrombopag is approved for treatment of thrombocytopenia in adult CLD patients undergoing elective invasive procedures in Japan, the USA, and the EU [2–4].

The pharmacokinetics of lusutrombopag is linear from 0.25 to 50 mg in single- or multiple-dose studies [3, 4]. The accumulation ratios of *C*_max_ and AUC were approximately 2 with once-daily multiple-dose administration, and steady-state plasma lusutrombopag concentrations were achieved after day 5 [3]. Lusutrombopag is mainly metabolized by CYP4 enzymes, including CYP4A11 [3, 4].

In vitro studies using transporter-expressing cells demonstrated that lusutrombopag was a substrate of P-glycoprotein (P-gp) and breast cancer resistance protein (BCRP), but not a substrate of organic anion transporter polypeptide (OATP) 1B1, OATP1B3, or organic cation transporter (OCT) 1 [3]. In vitro studies using human liver microsomes, hepatocytes, or transporter-expressing cells also assessed the inhibitory/inductive potential of lusutrombopag on human cytochrome P450 (CYP) enzymes and transporters, and demonstrated no or low inhibitory/inductive potential of lusutrombopag [3]. However, lusutrombopag exhibited time-dependent inhibition and lower 50% inhibitory concentration for CYP3A (8.8 μmol/L for midazolam 1′-hydroxylation) than that for other CYP enzymes (5.0 to > 75 μmol/L) (Shionogi data on file).

Based on the in vitro DDI study results, we conducted clinical drug-drug interaction (DDI) studies to evaluate the effect of lusutrombopag on the pharmacokinetics of midazolam, a CYP3A probe drug [5], and the effect of cyclosporine, which inhibits P-gp and BCRP [5, 6], on the pharmacokinetics of lusutrombopag in healthy subjects. Since the clinical DDI study assessing the effect of lusutrombopag on the pharmacokinetics of midazolam was conducted using a lower dose of lusutrombopag (0.75 mg) than the clinical dose used in the patients with CLD (3 mg) to avoid excessive platelet increase in healthy subjects, a physiologically based PK (PBPK) modeling was performed to evaluate the effect of lusutrombopag on the activity of CYP3A at the clinical dose of 3 mg in the target patient population.

## Methods

### Clinical studies

#### Ethics

This study was conducted under the principles of the Declaration of Helsinki. Written informed consent was obtained from each subject at the screening visit prior to the initiation of any study-related procedures. The investigator or designee explained the study procedures, risks, and potential benefits, if any. Subjects reviewed any study instructions and an informed consent form, and were given the time and opportunity to have any questions concerning the conduct of the study answered to their satisfaction. The investigators for both studies obtained Institutional Review Board approval for the protocol and all protocol amendments, and the written informed consent prior to study initiation.

#### Study design

In Study 1 (study 0912M0617), the potential inductive or inhibitory effect of lusutrombopag on the PK of midazolam, a CYP3A probe substrate [5], was assessed in a fixed-sequence drug interaction study in healthy adult subjects. A single 5-mg dose of midazolam (as syrup) was administered alone on day 1 under fasting conditions with 240 mL water, followed by administration of a 1.5-mg dose (six 0.25-mg tablets) of lusutrombopag on day 2, after which 0.75-mg doses (three 0.25-mg tablets) of lusutrombopag were administered once daily for 6 days (days 3 to 8) under fasting conditions. On day 8, a single 5-mg dose of midazolam was coadministered with the last dose of lusutrombopag under fasting conditions. Subjects who were enrolled in the DDI study with midazolam were admitted to the clinical research unit 2 days prior to the first dose of midazolam and remained in the unit for the 8-day dosing period and the 7-day follow-up period. Blood samples were collected and analyzed to determine the concentration of midazolam in plasma on days 1 and 8 immediately prior to dosing and at 0.5, 0.75, 1, 2, 3, 4, 5, 6, 8, 12, 16, and 24 h after dosing.

In Study 2 (study 1514M061E), the potential effects of cyclosporine, which inhibits P-gp and BCRP [5, 6], on the PK of lusutrombopag were assessed in a randomized, open-label, crossover study in healthy Japanese adult male subjects. Each subject was randomized to 1 of 2 treatment sequences in which they received a single 3-mg dose of lusutrombopag alone or coadministered with a single 400- or 600-mg dose of cyclosporine under fasted conditions in a crossover manner with a washout period of 21 days. The dose of cyclosporine was determined based on body weight: 400 mg for subjects weighing between ≥ 50 and < 65 kg, 500 mg for subjects weighing between ≥ 65 and < 75 kg, and 600 mg for subjects weighing between ≥ 75 and < 85 kg. Since the maximum dose of cyclosporin dose approved in Japan was 8 mg/kg per dose, the cyclosporine dose in this study was determined based on body weight as 400 to 600 mg. It was reported that the cyclosporine dose of 400 to 600 mg indicated in vivo inhibition via P-gp [7, 8]. Subjects were to receive lusutrombopag and quinidine (a selective P-gp inhibitor) as the third period, depending on the magnitude of increase in *C*_max_ and AUC of coadministered lusutrombopag compared with lusutrombopag alone in the first and second periods: if the upper limits of the 90% CIs for *C*_max_ and AUC exceeded 125%, the third period of the study would be carried out as planned. The magnitude of the increase with cyclosporine did not reach the prespecified criterion (1.25-fold increase in *C*_max_ and AUC) and thus, the third period was no longer required for the investigation. The above criteria for conducting the third period were prespecified in the protocol before starting the enrollment of subjects. In each period, blood samples for PK analysis of plasma lusutrombopag concentrations were collected predose and at 0.5, 1, 2, 3, 4, 5, 6, 8, 10, 12, 24, 36, 48, 72, 96, 120, and 144 h postdose.

#### Bioanalytical methods

A validated method using high-performance liquid chromatography–tandem mass spectrometry, which was reported previously [9, 10], was used to measure plasma concentrations of lusutrombopag.

#### Pharmacokinetic analysis

The following pharmacokinetic parameters were calculated based on the plasma concentrations of substrates (midazolam in Study 1 and lusutrombopag in Study 2) using non-compartmental analysis: maximum plasma concentration (*C*_max_), time to maximum plasma concentration (*T*_max_), area under the plasma concentration-time curve from time zero to the time of last quantifiable concentration after dosing (AUC_0–last_), area under the plasma concentration-time curve extrapolated from time zero to infinity (AUC_0–inf_), and terminal elimination half-life (*t*_1/2,z_). The pharmacokinetic parameters were calculated using Phoenix WinNonlin version 6.4 (Pharsight Corporation, St. Louis, MO).

#### Statistical analysis

An analysis of variance (ANOVA) was performed to compare pharmacokinetic parameters (*C*_max_, AUC_0–last_, and AUC_0–inf_) using Proc Mixed with SAS version 9.2 or 9.3 (SAS Institute Inc., Cary, NC, USA). The ln-transformed values of pharmacokinetic parameters were analyzed using an ANOVA model with terms for treatment, sequence, and period as fixed effects and subject within sequence as a random effect. The ANOVA was performed separately for midazolam pharmacokinetic parameters in Study 1 and lusutrombopag pharmacokinetic parameters in Study 2. The point estimates and 90% confidence intervals (CIs) were generated for differences between treatments. Based on the point estimates and 90% CIs, the corresponding geometric least squares (GLS) mean ratios and 90% CIs were calculated.

#### Safety assessment

In each treatment, safety evaluation assessed AEs, treatment-emergent AEs (TEAEs), vital signs, electrocardiogram findings, laboratory tests, platelet aggregation, and any other parameters that were relevant for safety assessment.

### Physiologically based PK modeling

Simcyp® Simulator version 14 release 1 (Certara USA, Inc., Princeton, NJ, USA) was used to simulate the DDIs. A PBPK model for lusutrombopag was built based on both preclinical and clinical data. The setting for input parameters of midazolam and virtual subject population are presented in Supplementary Information. For prediction of DDI potency at clinical dosage regimens of lusutrombopag, the model was employed to simulate plasma midazolam concentrations following single oral dosing of midazolam (5 mg) alone or with coadministration of lusutrombopag at oral 3-mg dose once daily for 14 days in the fasted state in healthy subjects. The simulations were performed for 100 subjects (10 trials and 10 subjects per trial) under each dose regimen of lusutrombopag (3 or 6 mg). *C*_max_ and AUC of lusutrombopag at 3 mg once daily in patients with CLD estimated in the population pharmacokinetic analysis [11] were 2-fold higher than those at 3 mg once daily in simulated healthy subjects in Simcyp. Therefore, 6 mg once daily for 14 days, which provided comparable exposure to 3 mg once daily in the target patients, was also tested in PBPK modeling to evaluate the effect of lusutrombopag at a 3-mg dose on CYP3A activity in the target patients.

## Results

### Clinical studies

#### Study 1: Effect of multiple doses of lusutrombopag on the pharmacokinetics of midazolam (a CYP3A substrate)

**Fig. 1:**
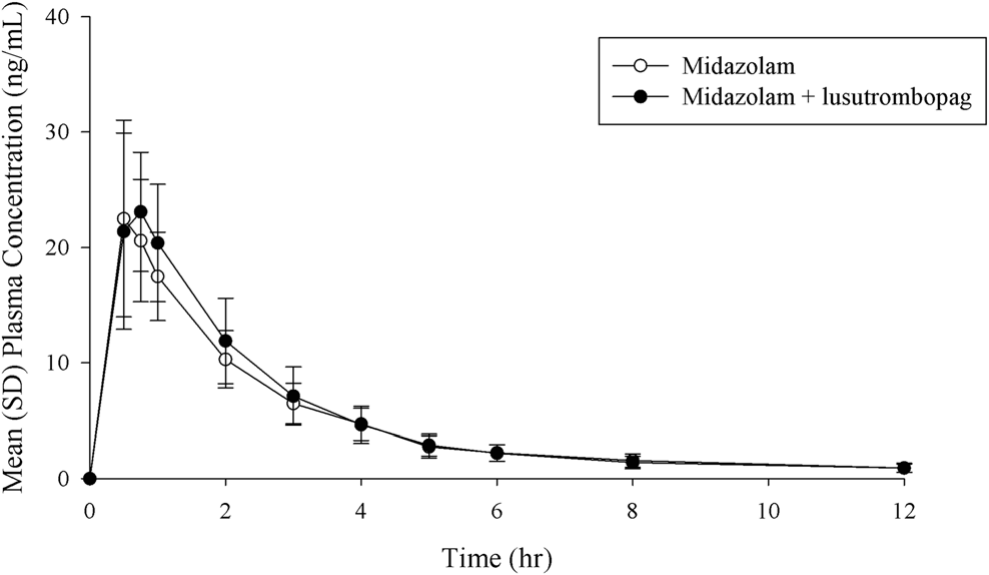
Mean (SD) plasma concentration profiles of midazolam following a single dose of midazolam 5 mg alone and with multiple doses of lusutrombopag. hr, hours; SD, standard deviation. 15 subjects/group

Thirteen of 15 subjects were male and the mean subject age was 34.9 years (Supplementary Table S1). Mean plasma concentration profiles of midazolam following the single dose of midazolam were similar between midazolam alone and coadministration with lusutrombopag (Fig. 1). No difference in the PK parameters of midazolam was noted between coadministration with lusutrombopag and administration of midazolam alone (Table 1), and the GLS mean ratios (90% CIs) of midazolam (coadministration with lusutrombopag to single administration of midazolam) in ANOVA were 1.01 (0.908–1.13), 1.04 (0.975–1.11), and 1.04 (0.967–1.11) for *C*_max_, AUC_0–last_, and AUC_0–inf_, respectively, suggesting no clinically significant effect of lusutrombopag on CYP3A activity.

**Table 1:** Pharmacokinetic parameters of midazolam following single dose of midazolam 5 mg alone and with multiple dose of lusutrombopag

Parameter (*N* = 15)	Midazolam alone	Midazolam + lusutrombopag	GLS mean ratio^a^ (90% CI)
*C*_max_ (ng/mL)	23.8 (25.1)	24.1 (27.7)	1.01 (0.908, 1.13)
*T*_max_ (h)^b^	0.50 (0.50, 1.00)	0.75 (0.50, 1.00)	-
AUC_0–last_ (ng h/mL)	58.7 (28.6)	61.0 (31.0)	1.04 (0.975, 1.11)
AUC_0–inf_ (ng h/mL)	63.0 (28.4)	64.9 (32.5)^c^	1.04 (0.967, 1.11)
*λ*_z_ (h^−1^)	0.165 (39.4)	0.186 (54.9)^c^	-
*t*_½,z_ (h)	4.21 (39.4)	3.74 (54.9)^c^	-
CL/F (L/h)	79.4 (28.4)	77.0 (32.5)^c^	-

-, not calculated; *CI*, confidence interval; *GLS*, geometric least squares

Geometric mean (% coefficient of variation) except for *T*_max_. Median (minimum, maximum) for *T*_max_

^a^GLS mean ratio (coadministration with lusutrombopag/midazolam alone)

^b^Median (minimum, maximum)

^c^*n* = 14

Overall, 8 subjects experienced at least 1 TEAE during the study. None of the TEAEs was reported as severe or serious. All of the TEAEs other than the sedation, which was reported by 2 subjects during the midazolam-alone dosing period, were considered unlikely to be related to lusutrombopag. No subject was withdrawn from the study due to a TEAE.

#### Study 2: Effect of a single dose of cyclosporine, which inhibits P-gp and BCRP, on the pharmacokinetics of lusutrombopag

**Fig. 2:**
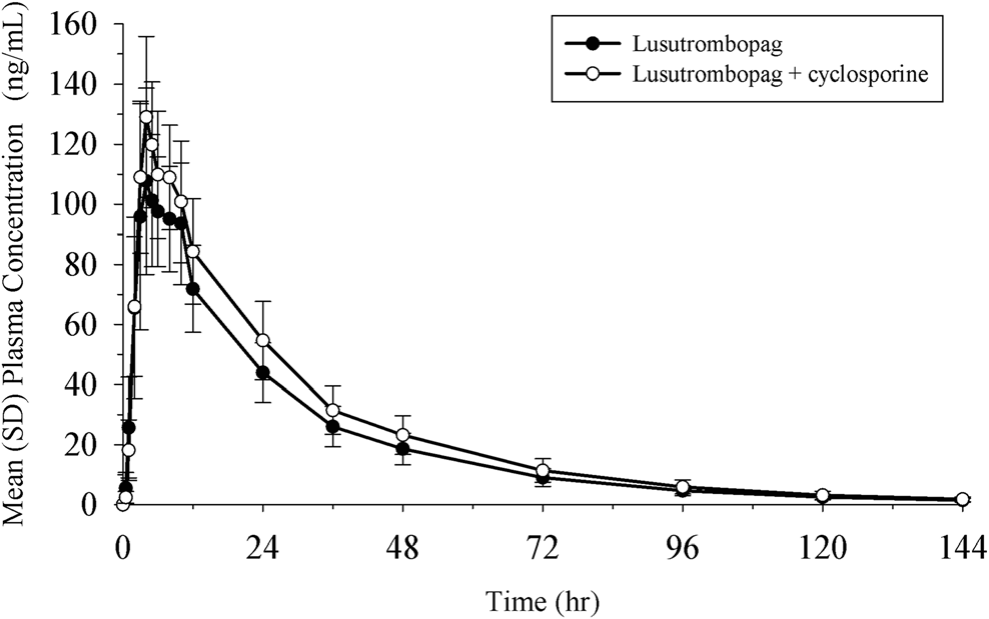
Mean (SD) plasma concentration profiles of lusutrombopag following a single dose of lusutrombopag 3 mg alone and with a single dose of cyclosporine. hr, hours; SD, standard deviation. 16 subjects/group

All 16 subjects were male and their mean age was 32.3 years (Supplementary Table S1). Mean plasma concentration profiles of lusutrombopag following a single dose of lusutrombopag were similar between lusutrombopag alone and coadministered with cyclosporine (Fig. 2). In ANOVA, the GLS mean ratios (90% CIs) of *C*_max_, AUC_0–last_, and AUC_0–inf_ of lusutrombopag coadministered with cyclosporine relative to lusutrombopag alone were 1.18 (1.11 to 1.24), 1.19 (1.13 to 1.25), and 1.19 (1.13 to 1.25), respectively (Table 2). Coadministration with cyclosporine thus increased lusutrombopag *C*_max_ by 18% and AUCs by 19% compared with lusutrombopag alone. The upper limit of the 90% CIs for *C*_max_ was < 1.25, and those for AUCs were 1.25. The point estimates of these increases were within a “default no-effect boundary” of 0.8 to 1.25 specified in the FDA DDI guidance [12], suggesting slight effect of coadministration with cyclosporine on the pharmacokinetics of lusutrombopag.

**Table 2:** Summary of pharmacokinetic parameters of lusutrombopag following single dose of lusutrombopag 3 mg alone and with single dose of cyclosporine

Parameter (*N* = 16)	Lusutrombopag alone	Lusutrombopag + cyclosporine	GLS mean ratio^a^ (90% CI)
*C*_max_ (ng/mL)	111 (20.4)	131 (18.0)	1.18 (1.11, 1.24)
*T*_max_ (h)^b^	4.00 (3.00, 8.00)	4.00 (4.00, 6.00)	-
AUC_0-last_ (ng h/mL)	2876 (23.2)	3426 (24.2)	1.19 (1.13, 1.25)
AUC_0-inf_ (ng h/mL)	2931 (23.4)	3491 (24.6)	1.19 (1.13, 1.25)
*λ*_z_ (h^−1^)	0.0259 (8.8)	0.0265 (9.2)	-
*t*_½,z_ (h)	26.8 (8.8)	26.2 (9.2)	-
CL/F (L/h)	1.02 (23.4)	0.859 (24.6)	-
V_z_/F (L)	39.5 (23.5)	32.5 (24.3)	-

-, not calculated; *CI*, confidence interval; *GLS*, geometric least squares

Geometric mean (% coefficient of variation) except for *T*_max_. Median (minimum, maximum) for *T*_max_

^a^GLS mean ratio (coadministration with cyclosporine/lusutrombopag alone)

^b^Median (minimum, maximum)

A total of 12 TEAEs were reported in 8 of the 24 subjects (33.3%) in the period of coadministration of lusutrombopag with cyclosporine, and no TEAEs were reported in the period of administration of lusutrombopag alone. All the TEAEs were considered not related to lusutrombopag by the investigator or subinvestigator. No deaths, SAEs, severe TEAEs, or AEs leading to withdrawal from the study were reported.

### PBPK modeling to evaluate effect of multiple doses of lusutrombopag at the clinical dose on CYP3A activity

The parameters of lusutrombopag in the PBPK model are shown in Supplementary Table 2. The median ratios (90% prediction intervals) of midazolam *C*_max_ and AUC following coadministration of midazolam with lusutrombopag compared with midazolam alone were 1.05 (1.02 to 1.12) and 1.05 (1.02 to 1.12), respectively, for lusutrombopag 3 mg; and 1.10 (1.04 to 1.23) and 1.10 (1.04 to 1.23), respectively, for lusutrombopag 6 mg (Table 3). For both dose regimens of lusutrombopag, the 90% prediction intervals for the ratios of midazolam *C*_max_ and AUC were within a “default no-effect boundary” of 0.8 to 1.25 specified in the FDA DDI guidance [12]. The simulations indicated no clinically significant DDI potential of lusutrombopag on CYP3A activity at the clinical dose (3 mg) in the target patients.

**Table 3:** Summary of simulation results of PBPK modeling for effect of lusutrombopag with multiple dose of 3 mg or 6 mg on pharmacokinetics of midazolam

Lusutrombopag dose	Parameter	Geometric mean (90% PI)		Ratio (with/alone)^a^
		Alone	With	
3 mg	*C*_max_ (ng/mL)	15.61 (6.36, 39.29)	16.57 (6.87, 40.58)	1.05 (1.02, 1.12)
	AUC (ng h/mL)	56.20 (16.46, 139.10)	59.67 (17.78, 143.50)	1.05 (1.02, 1.12)
6 mg^b^	*C*_max_ (ng/mL)	15.61 (6.36, 39.29)	17.32 (7.36, 41.55)	1.10 (1.04, 1.23)
	AUC (ng h/mL)	56.20 (16.46, 139.10)	62.38 (19.01, 146.77)	1.10 (1.04, 1.23)

*PI*, prediction interval

^a^Median (90% PI)

^b^The 6-mg dose was tested to achieve comparable exposure at the clinical dose of 3 mg once daily for 7 days in the target patients

## Discussion

In vitro, lusutrombopag inhibited the activity of CYP1A2, 2A6, 2B6, 2C8, 2C9, 2C19, 2D6, 3A4/5, and 4A11 in a concentration-dependent manner (Shionogi data on file). Using the mechanistic static model approach [13], the AUC ratio of each specific substrate was low (1.00 to 1.01) (Shionogi data on file). Lusutrombopag exhibited time-dependent inhibition of CYP3A and had lower 50% inhibitory concentration of CYP3A than those for other CYP enzymes. Therefore, the current clinical DDI study was conducted to assess in vivo inhibition potential for the CYP3A activity, given that there are many CYP3A substrate drugs on the market, although low inhibition had been expected. The result demonstrated that multiple-dose administration of lusutrombopag at 0.75 mg does not affect the pharmacokinetics of midazolam. The PBPK modeling suggested no clinically relevant effect of lusutrombopag at the clinical dose (3-mg dose) in the target patients on the pharmacokinetics of midazolam. Based on these results, lusutrombopag is unlikely to inhibit any of the evaluated enzymes at the clinical dose of 3 mg once daily.

Based on the in vitro studies, lusutrombopag was a substrate of P-gp and BCRP but not a substrate of OATP1B1, OATP1B3, or OCT1. The clinical DDI study demonstrated that coadministration with cyclosporine, which inhibits P-gp and BCRP, increased lusutrombopag *C*_max_ by 18% and AUC by 19% compared with lusutrombopag alone, suggesting a slight effect of cyclosporine on the pharmacokinetics of lusutrombopag. The role of P-gp and BCRP is not likely to be clinically significant in the intestinal absorption or biliary excretion for lusutrombopag. To further investigate the impact of the increased exposure with P-gp and BCRP inhibitor, a simulation of platelet counts using the reported pharmacokinetic/pharmacodynamic model [14] was conducted to estimate the probability of exceeding 200,000/μL platelets (platelet overshooting) with a 1.25-fold increase in lusutrombopag exposure (the upper limits of 90% CIs in Study 2). The probability of exceeding 200,000/μL platelets with the increased lusutrombopag exposure was still low (1.12% with a 1.25-fold increase in exposure and 0.43% with no increase) (Shionogi data on file). Based on these results, it could be concluded that there would be no clinically significant effect of P-gp and/or BCRP inhibitors on the pharmacokinetics of lusutrombopag.

A 3-mg dose once daily for 7 days is approved for treatment of thrombocytopenia in patients with chronic liver disease. However, a clinical study was conducted to assess an effect of lusutrombopag on the pharmacokinetics of midazolam with a 0.75-mg once-daily dose (1.5-mg loading dose). This is because it was ethically difficult to conduct a clinical DDI study with 3-mg multiple dose of lusutrombopag in healthy subjects, since the platelet counts remarkably increased in all subjects who received the 2-mg multiple dosing, suggesting the multiple dosing of clinical dosage (3 mg) would not be allowable for a DDI study in healthy subjects from a safety point of view. In this case, in silico PBPK modeling approach would be useful in predicting the inhibition potential of lusutrombopag at the clinical dose, based on the clinical DDI study result with lower dose (0.75-mg once daily), although the limitation of extrapolation to a higher dose should be noted.

Another limitation of these DDI studies was the limited number of female subjects enrolled (2 females in Study 1 and no female in Study 2). However, to our best knowledge, clinically significant sex differences in DDI via P-gp or BCRP have not been reported. In Study 1, the ratios of midazolam *C*_max_ and AUC_0–inf_ for coadministration with lusutrombopag relative to midazolam alone were 1.23 to 1.29 and 1.08 to 1.12, respectively, in the 2 female subjects, which were within the range of those in male subjects, supporting no sex difference in the DDI potential.

Plasma concentrations of lusutrombopag were measured on days 3 to 8 after multiple doses in Study 1, and plasma concentrations of cyclosporin were measured after a single dose in Study 2. It was confirmed that they were consistent with those reported in the previous reports, supporting the adequate exposure for evaluating the inhibitory effects.

The majority of the subjects were white in Study 1, and all of the subjects were Japanese in Study 2. However, there was no clinically significant ethnic difference in the pharmacokinetics of lusutrombopag in the population pharmacokinetic analysis [14]. In addition, no clinically significant difference in CYP3A4 activity was reported between Japanese and European American subjects [15]. The geometric mean *C*_max_ and AUC_0–inf_ of cyclosporine were 1770 ng/mL and 10,640 ng h/mL, respectively, in Japanese subjects in Study 2. The pharmacokinetic parameters of cyclosporine were similar to those after a single dose of cyclosporine 400 mg reported in other ethnicities (19 white and 1 black subjects; 1516 ng/mL and 8631 ng h/mL for *C*_max_ and AUC_0–inf_, respectively) [8]. Therefore, the results in Study 1 and Study 2 were considered to be extrapolated to other ethnicities.

Lusutrombopag is mainly metabolized by CYP4 enzymes, including CYP4A11 [3, 4]. Drug interactions mediated by inhibition or induction of any CYP4 enzymes have not been reported in clinical use. Therefore, inducers and inhibitors of CYP4 enzymes including CYP4A11 (e.g., clofibrate as an inducer of CYP4 enzymes [16]) are unlikely to affect the pharmacokinetics of lusutrombopag.

In vitro studies demonstrated no induction effect of lusutrombopag on the activity of CYP1A2, 2C9, 3A4, UGT1A2, 1A6, and 2B7. Lusutrombopag also had low potential to inhibit P-gp, BCRP, OATP1B1, OATP1B3, OCT1, OCT2, OAT1, OAT3, MATE1, MATE2-K, and BSEP.

Cyclosporine has inhibitory effects on OATPs and CYP3A enzymes as well as P-gp and BCRP. However, lusutrombopag is a substrate of P-gp and BCRP, but not of other transporters. In addition, lusutrombopag is metabolized primarily by CYP4 enzymes. Therefore, it was considered that lusutrombopag and cyclosporine were mechanistically interacted via P-gp and/or BCRP.

In conclusion, based on the in vitro, in vivo, and in silico results, no clinically significant DDI potential of lusutrombopag with other medical products via metabolic enzymes and transporters is suggested.

## Electronic supplementary material

ESM 1 (DOCX 29 kb)
