# Supplementary material for: Evaluation of drug-drug interaction of lusutrombopag, a thrombopoietin receptor agonist, via metabolic enzymes and transporters
Source: Eur J Clin Pharmacol. 2020 Jul 14;76(12):1659–65. doi: 10.1007/s00228-020-02960-7 (PMC7661413; doi:10.1007/s00228-020-02960-7)
Supplement: Supplementary file 1 — (DOCX 29 kb) [file 228_2020_2960_MOESM1_ESM.docx]

**Supplementary materials**

**Supplementary Table S1** Demographics of study subjects

| Variable | Study 1 | Study 2 |
| --- | --- | --- |
| Number of subjects | 15 | 16 |
| Age (years)^a^ | 34.9 (24, 55) | 32.3 (21, 46) |
| Sex |  |  |
| Male | 13 | 16 |
| Female | 2 | 0 |
| Race |  |  |
| Asian | 1 | 16 |
| Black or African American | 3 | 0 |
| White | 11 | 0 |
| Body weight (kg)^a^ | 81.5 (64.7, 108.8) | 68.7 (55.0, 78.7) |
| Body mass index (kg/m^2^)^a^ | 26.7 (21.3, 31.1) | 24.0 (18.6, 28.8) |

^a^ Mean (range)

**Supplementary Table S2** Input parameters of lusutrombopag

| Input parameter | Value | Comment/source |
| --- | --- | --- |
| *Physical chemistry and blood binding* | | |
| MW | 591.5 | In-house data |
| LogP | 8.64 | In-house data |
| Compound type | Diprotic acid | In-house data |
| pKa | 3.52, 6.52 | In-house data |
| B/P | 0.55 | Minimal distribution to red blood cell |
| f_u_ | 0.00004 | In-house data |
| Plasma binding component | Human serum albumin | Assumed |
| *Absorption* | | |
| Absorption model | 1st-order model |  |
| f_a_ | 1 | Assumed |
| k_a_ (hr^-1^) | 0.426 | Estimated from data in clinical study |
| CV% for k_a_ | 30 | Assumed |
| lag time for absorption (hr) | 0 | Assumed |
| f_u,gut_ | 1 | Assumed |
| Q_gut_ (L/hr) | Predicted^a^ | Caco-2 permeability of 0.258×10^-6^ cm/sec |
| *Distribution* | | |
| Distribution model | Minimal PBPK model |  |
| V_ss_ (L/kg) | 0.495 | Estimated from data in clinical study |
| CV% for V_ss_ | 30 | Assumed |
| Kp liver | 1 | Assumed |
| *Elimination* | | |
| Clearance type | In vivo clearance |  |
| CL_po_ (L/hr) | 1.39 | Estimated in clinical study |
| CL_R_ (L/hr) | 0 | Low urinary excretion |
| Active uptake into hepatocyte | 1 | Assumed^b^ |
| *Interaction for CYP3A4* | | |
| k_i_ (µM) | 4.5 | Estimated from data in *in vitro* study |
| f_u,mic_ for reversible inhibition | 1 | Assumed^c^ |
| k_inact_ (hr^-1^) | 54.7 | Estimated from data in *in vitro* study |
| k_app_ (µM) | 1000 | Estimated from data in *in vitro* study |
| f_u,mic_ for MBI | 1 | Assumed^c^ |

*MW*, molecular weight; *LogP*, logarithmic octanol:water partition coefficient; *pKa*, negative logarithmic acid ionization constant; *B/P*, blood to plasma partition ratio; *f_u_*, fraction of drug unbound in plasma; *f_a_*, fraction of drug absorbed; *k_a_*, absorption rate constant; *CV*, coefficient of variation; *f_u,gut_*, fraction of drug unbound in gut; *Q_gut_*, nominal flow rate of drug to gut; *V_ss_*, volume of distribution at steady state; *Kp liver*, liver/plasma partition coefficient; *CL_po_*, oral clearance; *CL_R_*, renal clearance; *k_i_*, reversible inhibition constant; *f_u,mic_*, fraction of drug unbound in microsomes; *k_inact_*, maximum inactivation rate of the enzyme; *k_app_*, concentration given half of maximal inactivation rate; *MBI*, mechanism-based inhibition

^a^ Apical pH:basolateral pH, 7.4:7.4; activity: passive & active

**Supplementary Information** Setting for input parameters of midazolam and virtual subject population in physiologically-based pharmacokinetic modeling

(a) Setting for input parameters of midazolam

“Sim-Midazolam (version 14 release 1)” of the Simcyp compound library was used for setting input parameters of midazolam except for k_a_. Midazolam k_a_ was changed to 8.78 hr^-1^ as the estimate based on the plasma concentration data of midazolam in Study 1, using the Parameter Estimation function in Simcyp.

(b) Setting for virtual subject population

Healthy male Caucasian subjects 20-64 years old were simulated using the Population file “Sim-Healthy Volunteers (version 14 release 1)” of the Simcyp compound library.
